# Perspective on optimizing clinical trials in critical care: how to puzzle out recurrent failures

**DOI:** 10.1186/s40560-016-0191-y

**Published:** 2016-11-04

**Authors:** Bruno François, Marc Clavel, Philippe Vignon, Pierre-François Laterre

**Affiliations:** 1 Medical-surgical Intensive Care Unit, Dupuytren University Hospital, 2 avenue Martin Luther King, 87042 Limoges, France; 2 Inserm CIC 1435, Limoges University Hospital, Limoges, France; 3 Medical-surgical Intensive Care Unit, Cliniques Saint-Luc, Brussels, Belgium

**Keywords:** Clinical research, Intensive care unit, Trials, Ventilator-associated pneumonia, Investigation center, Performance

## Abstract

**Background:**

Critical care is a complex field of medicine, especially because of its diversity and unpredictability. Mortality rates of the diseases are usually high and patients are critically ill, admitted in emergency, and often have several overlapping diseases. This makes research in critical care also complex because of patients’ conditions and because of the numerous ethical and regulatory requirements and increasing global competition. Many clinical trials in critical care have thus failed and almost no drug has yet been developed to treat intensive care unit (ICU) patients. Learning from the failures, clinical trials must now be optimized.

**Main body:**

Several aspects can be improved, beginning with the design of studies that should take into account patients’ diversity in the ICU. At the site level, selection should reflect more accurately the potential of recruitment. Management of all players that can be involved in the research at a site level should be a priority. Moreover, training should be offered to all staff members, including the youngest. National and international networks are also part of the future as they create a collective synergy potentially improving the efficacy of sites. Finally, computerization is another area that must be further developed with the appropriate tools.

**Conclusion:**

Clinical research in the ICU is thus a discipline in its own right that still requires tailored approaches. Changes have to be initiated by the investigators themselves as they know all the specificities of the field.

## Background

Critical care is probably one of the most complex fields of medicine and has specific requirements. Severely ill patients who are hospitalized in the intensive care unit (ICU) require continuous monitoring and management and the presence of attending physicians 24/7, as opposed to most medical specialties with intermittent care [1]. Clinical research in the ICU setting is challenging because of an overall high mortality rate, the high number of healthcare workers including physicians with repeated rounds, the technological environment, and the unpredictable patients’ course with potential sudden worsening of clinical status. In addition, ICU patients typically sustain life-threatening conditions with multiple organ failures and various underlying diseases. ICU admission is unpredictable and eligible patients present with various types of diseases, even in specialized ICUs (neurological, cardiovascular, trauma…).

Even if some ICU-specific diseases have been described such as sepsis, acute respiratory distress syndrome (ARDS) or ventilator-associated pneumonia (VAP), most of them remain identified as syndromes [2] with lack of specificity, as illustrated by regular updates of the definitions [3–5]. Accordingly, even when selected through strict inclusion/exclusion criteria within a trial, study population remains heterogeneous. Finally, ethical issues inherent to the severely ill patients include the discussion of care withdrawal in specific settings and the frequent inability of ICU patients to consent [6, 7].

Critical care is also a relatively recent field of medicine and even if a lot have been accomplished, as mentioned by Takala, we are still in a learning phase for ICU-specific research development and especially for pharmaceutical-sponsored trials [8].

Accordingly, clinical trials have to be optimized both in terms of scientific approach and design. This perspective paper aims at understanding the current context of clinical trials in the ICU and its increasing difficulties and proposing solutions in terms of research organization and management.

## Main text

### Context

#### Increasing trial complexity

**Fig. 1:**
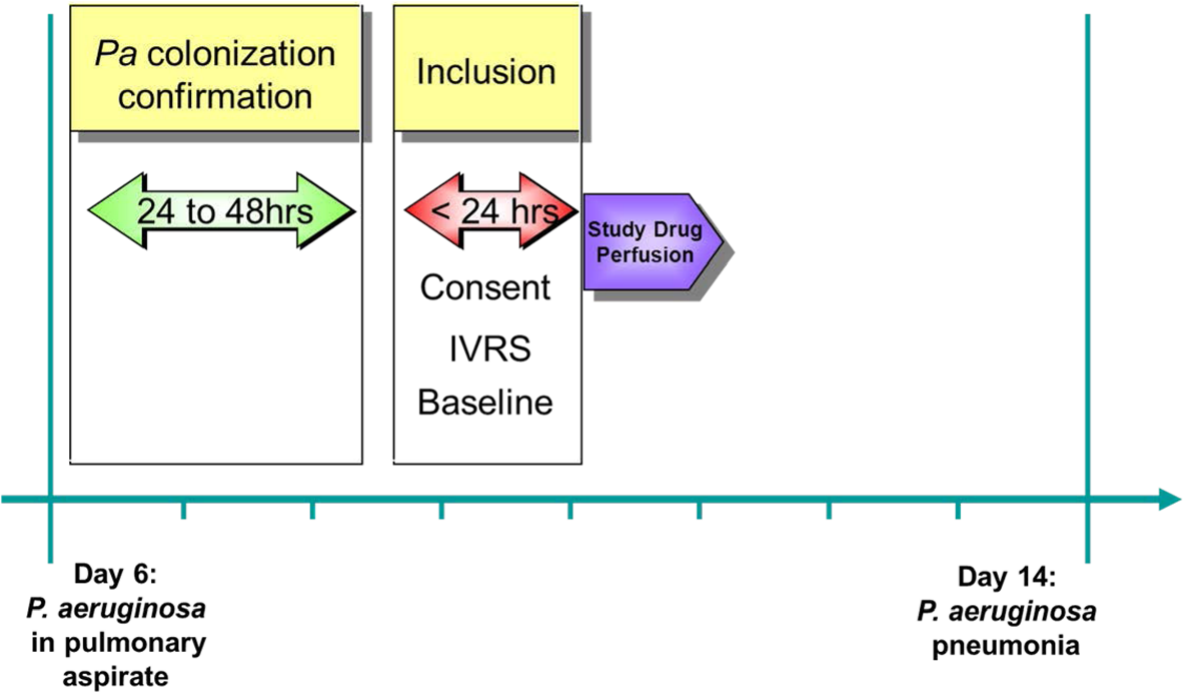
Example of a “mobile short time window” for enrolment in a pre-emptive approach trial targeting mechanically ventilated and colonized patients with *Pseudomonas aeruginosa* and before onset of VAP. *IVRS* interactive voice response system (randomization system)

Within a decade, trial complexity has dramatically increased. In order to target the most appropriate study population, the inclusion/exclusion criteria have increased and become more precise (e.g., severity of sepsis and number and type of associated organ failures) [9]. This increased complexity associated with narrow time windows for patients’ enrolment result in a time consuming and quite challenging screening process (Fig. 1). Pharmacokinetic explorations have also been added to most ICU randomized clinical trials (RCT). Finally, biomarkers are frequently used to better characterize the study population and increase the complexity of clinical research [10]. Some of them are commonly used, such as procalcitonin [11], but others such as HLA-DR result in a much more complex approach [12]. With the continuous progression of scientific knowledge, further complexity of future ICU clinical trials can be anticipated.

#### Increasing regulatory requirement

In order to address increasing safety concerns to protect the patients, strict regulations of clinical research have been developed. Between the last war when ethical rules have been proposed [13] and the early eighties, a few regulatory texts have been written. The International Conference of Harmonization (ICH) [14] was the very first text to define a global regulatory approach (first presentation in April 1990 in Brussels). Good clinical practice (GCP) [15] is probably the best example of a universal regulatory requirement but remains the minimal standard to perform clinical research. In addition, legal requirements exist for each component of clinical research, especially in drug development trials, including data management, informed consent [16], biological sampling, case report form (CRF)…. In some countries, national specificity still exists, such as the German *Bundesamt für Strahlenschutz* (Federal Office for Radiation Protection), a specific committee which evaluates the need of additional radiography or of CT scan prescription in clinical research projects to avoid useless detrimental radiation.

Thus, the regulatory work package has become central in clinical research and is often long to address, irrespective of the sponsor (academic or pharmaceutical). Because of its specificity, clinical research in critical care medicine has even more requirements since patients with life-threatening conditions are commonly unable to consent [17]. Emergency consent through legal representative or even waivers have been authorized to address this issue [18]. Agencies are currently paying particular attention to regulatory aspects with increasing control through audit or inspections [19], especially in high recruiting centers.

#### International competition

Clinical research has become an international competition and represents a strategic activity at a site level as well as at a country level [20]. Due to the financial investment required for the development of new drugs, there is a worldwide competition between pharmaceutical companies to obtain the leadership in specific medical fields. A similar competition is observed in the academic area to become and remain a key player in clinical research and also because it constitutes a strong economic vector through allocated budgets, royalties, patents, grants…. While it was mostly a Northern American and Western European activity few decades ago, most of developed countries are now involved in clinical research. This increases the international competition to actively participate in RCTs, with some countries which are now offered to participate in research projects, or with a much lower number of sites. In pharmaceutical-sponsored trials, the competition is even initiated by the companies themselves or the clinical research organizations (CRO) to accelerate completion of the trial.

#### Inappropriate trial design resulting in absence of “ICU-specific” drug

Critical care remains one of the leading medical specialties in the conduction of pathophysiological and epidemiological studies and has validated several severity scoring systems [21, 22]. Nevertheless, and since Xigris® (Lilly, Indianapolis, IN) withdrawal, it is also one of the medical specialties in which most clinical trials have failed with nearly none or very few drugs specifically developed and validated for ICU patients [23] despite strong scientific rational and encouraging results on animal models. To date, the clinical phase of trials seems to be the main issue. Patients are included based on precise clinical criteria such as organ failure, but therapeutic criteria are not given enough attention. Moreover, monitoring of the drug activity and outcomes may not be sufficient to adequately evaluate the study drug. Diagnostic companions and biomarkers are thus needed to better assess new drugs as this weakness may have led to drop the development of appropriate molecules. In addition, the unmet medical need in critical care medicine is probably one of the most important when considering the high mortality rate of prevalent diseases leading to ICU admission even if a slight decrease have been recently evidenced in sepsis mortality [24]. Despite recurrent failure of RCTs testing the efficacy of new drugs, clinical trials are still designed to find the “golden success bullet,” especially in the field of sepsis.

### Proposed solutions

#### Scientific challenges to be addressed

##### How to learn from our failures

Sepsis is a fascinating field in critical care medicine clinical research. After 25 years and >30,000 patients enrolled in successive clinical trials, not a single agent has yet been approved in this indication. The most recent RCTs were negative (e.g., TLR4 LPS antagonist) [25] or even early terminated (recombinant lactoferrin) despite strong scientific rational and promising early phases. Similarly, ventilation strategies (e.g., low tidal volume, prone position) rather than specific drug development have been shown to be successful in ARDS [26, 27]. VAP might thus remain the most successful disease of critical care medicine in terms of drug development as several antibiotics have been validated in this indication [28, 29]. We need to understand the reasons for so many failures and how to puzzle them out. “Back to basics” remains a good approach since the understanding of pathophysiology has to be revisited before moving into clinical applications [30]. Results interpretation especially in early clinical phases should be less “emotional.” Repeated phase II or II/III should also be encouraged in order to confirm results before implementing new drugs on clinical grounds [31].

When compared with oncology or even cardiology, most critical care diseases (VAP being one of the best examples) suffer from imprecise definition and lack of specific biomarkers. This results in heterogeneous study populations in which the demonstration of the efficacy and tolerance of a new drug or management strategy remains challenging. Accordingly, active research on specific biomarkers should be developed and once a biomarker is evidenced, it should be used as much as possible in the ICU setting to improve study population homogeneity [32]. Biomarkers can be considered as a diagnostic companion and part of the *theranostic* approach of a drug development.

Inclusion criteria must probably also be modified. As an example in sepsis trials, within a type of infection, different clinical phenotypes can exist depending on the pathogen (e.g., community-acquired pneumonia), and therefore, pathogen- or site-specific trials should be considered. Despite robust inclusion/exclusion criteria, large trials may also pool heterogeneous patients with limited discriminating data between subgroups with detrimental effects of studied drug. Accordingly, sample size could be redefined based on significance and on clinical relevance of endpoints.

It has also been demonstrated that the identification of eligible patients based on number of protocol violation improves in parallel with the number of patients enrolled in a trial. In some trials, violation of inclusion/exclusion criteria reached 20% in sites with low recruitment performance, whereas it fell to 5% in high recruiters. In fact, it seems that sites are following a research learning curve at every trial level [33].

##### Trial design (including personalized medicine)

For a long time, placebo-controlled, double-blind, multicenter randomized trials have been considered as the gold standard of clinical research in the ICU with the dictatorship of the *p* value. ICU patients have numerous concomitant overlapping diseases, when compared to patients of other medical specialties [34]. Accordingly, proving the benefit of a unique drug or of a specific intervention becomes challenging and endpoints aside from mortality are always controversial as they remain less relevant. Nevertheless, the need for clinical trials in the ICU has never been so high since most of the standard of care remains based on bundles or low-grade recommendations with a high heterogeneity.

Therefore, time for innovative design is becoming a reality to be able to adapt our trials to critical care medicine. Adaptive design is an appealing approach. It enables to consider several interventions, pharmacological or not, at the same time and to eliminate the less relevant ones as patients are progressively enrolled [35]. In order to take into account inter-site variability, the cluster approach is also one methodological design to consider for the ICU setting. Even if critical care medicine remains far behind oncology for example, personalized medicine will probably be the most interesting paradigm shift in the coming years. Patients are not considered anymore as similar and homogenous within a specific disease. Accordingly, specific trial designs and approaches will have to be developed for critically ill patients. Some other interesting approaches might also be developed for rare diseases as those caused by multiresistant pathogen in the ICU. In this case, random trials comparing similar patients receiving drugs from different pharmaceutical companies could be the most innovative design.

#### Site selection

Even if the quality of the study protocol and of the tested intervention (drug, device, or other type of therapeutic approach) are probably the two most important factors to make a trial successful, research sites remain key players in patient recruitment. Therefore, particular attention should be paid to site selection. Even if it has improved over the past decade, it remains highly perfectible especially in the field of critical care medicine. The traditional 20/80 research rule in critical care medicine illustrates the weakness of research site selection. In most multicenter studies, some sites include many patients while other centers include only a few or even none. Thus, the majority of patients (around 80%) are recruited by the same sites (accounting for 20% of all the sites involved in the study). Site selection is frequently based on inaccurate investigators listing [36]. Some of these lists are issued by the sponsor for non-scientific reasons and include key opinion leaders not necessarily involved on clinical grounds. Most of the potential investigators are provided by the CROs based on previous collaborations but frequently these lists have not long been updated or are not even adapted to the trial. Finally, some investigators can also be recommended by the principal investigator for personal reasons. Accordingly, optimization of trial delivery starts with a better site selection.

##### Feasibility process

The feasibility process mainly based on declarative information is a key step in site selection [37]. Most of the feasibility questions remain focused on site resources, contracting, and GCP training and not on the actual recruitment capacity. In addition, items focused on the targeted population are usually vague asking the investigator for potential recruitment capacity. Investigators frequently overestimate their recruitment capacity in order to be selected, and CRO competition can also lead to further overestimation. Feasibility which is usually assessed far ahead in the selection process should specifically focus on: (1) actual recruitment capability and (2) prior performance of the site in the same topic of research. Actual recruitment capacity can be accurately estimated by recording retrospectively or prospectively over 1 or 2 months (or more in case of seasonality of the studied disease) the actual number of eligible patients based on inclusion and exclusion criteria. Each site should be able to record exhaustively all eligible patients for a trial project over a predefined period of time. This will improve the chance to successfully complete the trial but also guide the site in its decision to participate or not in the research project. Usually, investigators have a biased estimation of their patient flows when going through screening processes, since they commonly fail to take into account time window constraints or potential exclusion criteria. Prior performance is the second key factor for site selection. The ratio between the number of predicted enrollments and the number of patients finally included in a trial is a commonly used indicator to assess the recruitment performance of a research site. A site that was efficient in previous trials is probably well organized and has enough human resources to plug clinical research on routine patient flow. The last key factor is the absence of potential competing trials during the same period. However, if high recruitment capacity enables adequate conduction of competing trials, a local randomization table between trials is recommended to avoid any recruitment bias. Such an integrative approach will strengthen site selection and potentially allow reducing the number of sites needed to complete the trial.

##### Networking

Networking may positively impact RCT delivery in critical care medicine. Since the development of multicenter RCTs, participating sites have developed informal networking. With the increasing requirements of clinical research, networking has become much more formal through charters in specific medical fields. Networks have been progressively structured based on scientific collaborations and on synergy between research sites, sharing standard operating procedures and research tools to better reach common objectives. Among medical specialties, oncology is probably the most advanced in networking since most of RCTs are delivered through structured networks [38]. This approach makes trial process and delivery much more efficient and faster. Success is not only related to the active participation of all identified sites but also to the input of network coordinators and the collective synergy created by the network. Efficient networks have fully demonstrated their efficiency in the academic field of critical care medicine through the completion of numerous successful trials, including the ANZICS [39, 40] and ARDS networks [26]. In addition, well-structured network with a strong experience in pharmaceutical-sponsored trials may positively impact recruitment. As an example, CRICS, a French clinical research network, has contributed to 35% of the global recruitment of a very challenging sepsis phase II trial while representing only 15% of participating sites [41]. In addition to their recruitment capability, research networks may participate to the homogeneity of patient management through similar standard operating procedures. In addition, such network may facilitate the feasibility process when performing network-centralized feasibility. In the near future, networking will probably become a pillar of RCT optimization in critical care medicine. Accordingly, many countries are currently establishing clinical research network certifications.

#### Site management

Once site selection is performed, the optimization of trial recruitment also relies on site management. It has long been mainly based on principal investigators with little attention paid to their environment. Understanding that RCT must be considered as a team activity brings in light that local site management is another pillar of clinical research.

##### Study team management

More than any other medical specialties, critical care medicine is based on a team activity for daily routine care. Regardless of the organization model, all staff members (physicians, fellows or residents, and nurses) are collectively in charge of patients, based on 24/7 shifts [1]. The same team approach has to be implemented in clinical research. Accordingly, all doctors and nurses should be involved in research activity, from patient enrollment to follow-up. Such as in oncology and hematology, all ICU care providers should incorporate clinical research as part of their daily routine care. Nevertheless, it is true that in many ICUs, physicians are overstrained with the routine clinical practice and sometimes do not even have enough time to complete all essential tasks. Accordingly, different solutions can be proposed to address this challenge depending on local organization specificities. The easiest one is probably to have at least study nurses available 24/7 to help doctors with inclusions. A more global approach, such as a dedicated research physician on call, can be proposed at very experienced place with enough medical resources. A unique investigator cannot be fully efficient in the conduction of a research trial, even with the help of study coordinators. Every healthcare worker of the ICU has to be involved in the investigation activity to ensure research continuity. Therefore, before acceptance of any new protocol, a common agreement of all medical staff is needed. In order to ensure the motivation and commitment of all the staff, specific training must be developed at the site level. Communication is also a key factor within the team and research meetings should be encouraged on a regular basis. Study team management must result in a positive collaboration and encourage each staff member to be dedicated to the trial. Leadership is therefore needed since individual approaches may generate conflicting situations and negatively impact the study. The leader has to develop some strong management capability in order to achieve this goal.

##### Site interaction

Regardless the type of ICU (surgical and/or medical, closed or open), they constitutively have transversal medical activities and receive patients from various clinical settings with a large panel of diseases. Accordingly, patient care requires interaction with numerous physicians and consultants but also easy and continuous access to technical platforms such as biology, microbiology, or radiology [42]. Thus, to ensure the appropriate participation of all these persons in clinical research, specific site interactions have to be developed through standard operating procedures. For example, close interactions with the microbiology department is crucial for sepsis or VAP trials. Indeed, most of the ICU trials on infectious diseases require microbiological availability at least 7/7, sometimes 24/7, using real-time techniques such as polymerase chain reaction. Involvement and valorization of these collaborators must be strengthened for a specific study but also more continuously with true interaction, routine common meeting, specific communications…. Pharmacy is another key actor of RCTs that has to work hand in hand with the ICU investigators and must not be considered as a simple “drug provider” [43]. At the ICU level, research is generally delivered by the study team including investigators, study nurses, and study coordinators. Nevertheless, interaction with the bedside nurses is key, these latter being patients’ main interlocutors and so potentially considered as a relay at bedside to ensure good implementation of the study. Finally, collaboration with all other hospital departments involved in intra-hospital patient course is essential to ensure full commitment to the clinical research activity. It is important for the recruitment period but also for patient follow-up (including diagnosis of adverse events for example) when they are discharged from ICU. For research in critical care medicine, the emergency department from where many patients are referred to the ICU is probably one of the most important partners. It takes often years to develop strong hospital interaction but every players inside or outside the ICU should be considered as a potential study team member or at least part of the research activity. Conflicting situations can result in absence of such fruitful collaboration.

#### Recruitment strategy

Recruitment is a potential area of optimization of RCTs [44]. Despite robust pre-study calculation and estimation of study population size, under-recruitment remains an important issue in trials conducted in ICU setting. For decades, the recruitment rate of most participating centers to therapeutic trials in critical care medicine has remained fairly low without true learning from failures and efficient corrective actions. The fact that patient flow is fully unpredictable in the ICU even during closed hours may partially explain enrolment difficulties. Nevertheless, some sites are used to respect their commitment regardless their size while some other frequently overestimate their recruitment capacity, demonstrating that recruitment strategy is probably one piece of the puzzle of success [45]. This strategy must be general at the site level including involvement of every ICU physicians participating in patient care, systematic screening upon admission and during ICU stay, “helping resources” 24/7 making investigator life easier, screening binders for all trials…It has also to be study specific and recruitment capability should be carefully evaluated since the very beginning of the project (see feasibility paragraph). Thus, for every new trial, special attention should be paid to identify the key determinant of recruitment based on inclusion/exclusion criteria and where and when patients will be into the eligibility target, in order to set up specific study recruitment organization.

Quality of recruitment can also be improved and the use of a clinical coordinating center (CCC) can be one of the most efficient solutions. The function of a CCC is to facilitate testing of new interventions, develop high-quality protocols for the conduct of phase II and phase III clinical trials, generate consistent interpretation of enrolment criteria, and ultimately allow the strict respect of research protocols [46]. It is currently widely used in ICU trials.

While clinical research has greatly improved in the past decades in the ICU, recruitment may remain the most perfectible weak point and sites should work on specific strategies.

#### Tools development

**Fig. 2:**
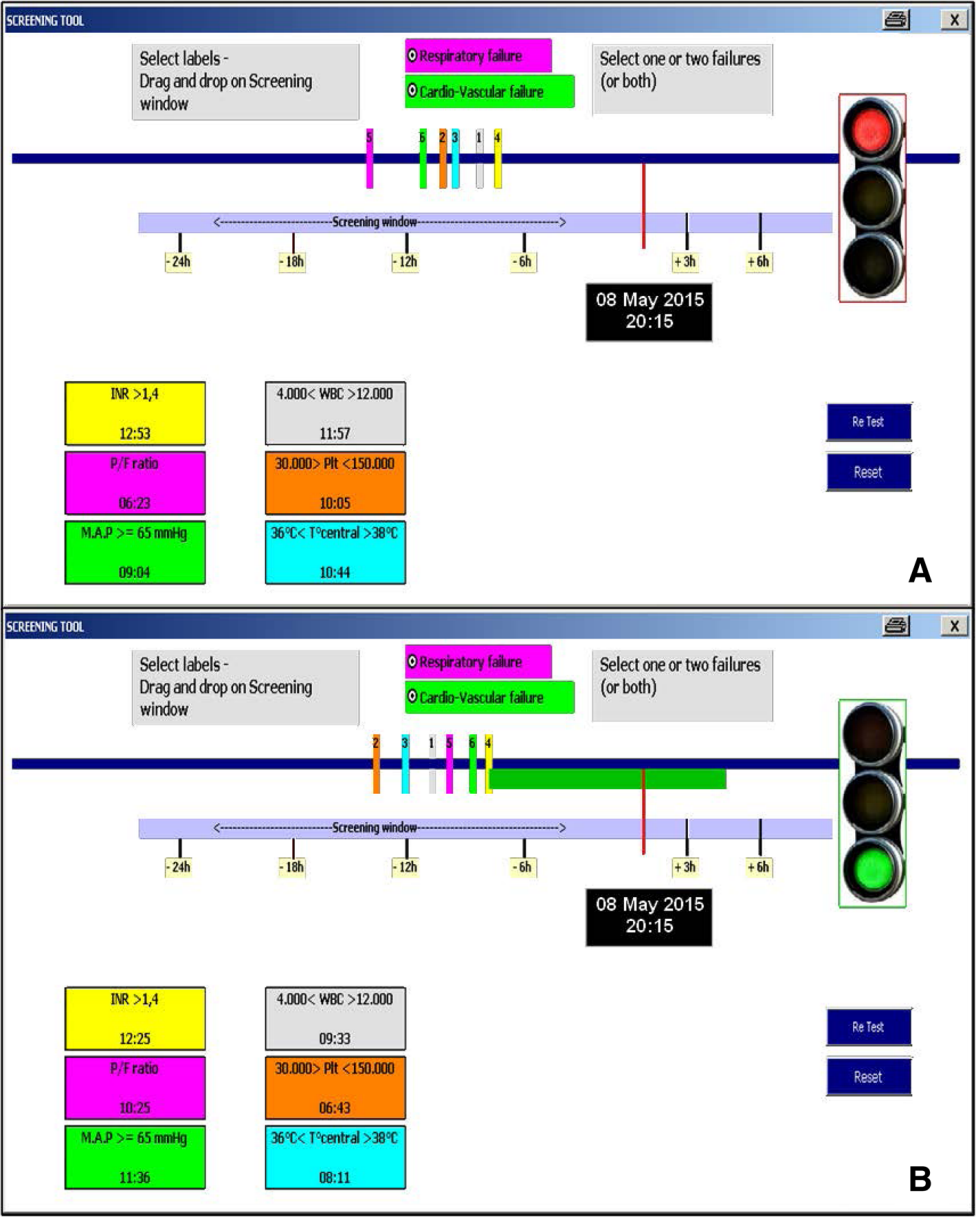
“Drag and drop” screening tool for eligibility checking in a sepsis trial with a narrow time window for enrolment. Each *numbered bar* represents a qualifying criterion. Whenever a criterion is recorded, the corresponding *bar* is dragged on the time line (*blue*). In fig. 2a, all the criteria were not recorded in the pre-specified window (P/F ratio recorded outside the time window). Thus, the patient cannot be included and the light is *red*. In fig. 2b, the light turned green as all criteria were recorded in the right time window and the time remaining for enrolment is indicated with the green bar. *INR* international normalized ratio, *MAP* mean arterial pressure, *WBC* white blood cells, *PLT* platelets, *T* temperature

**Fig. 3:**
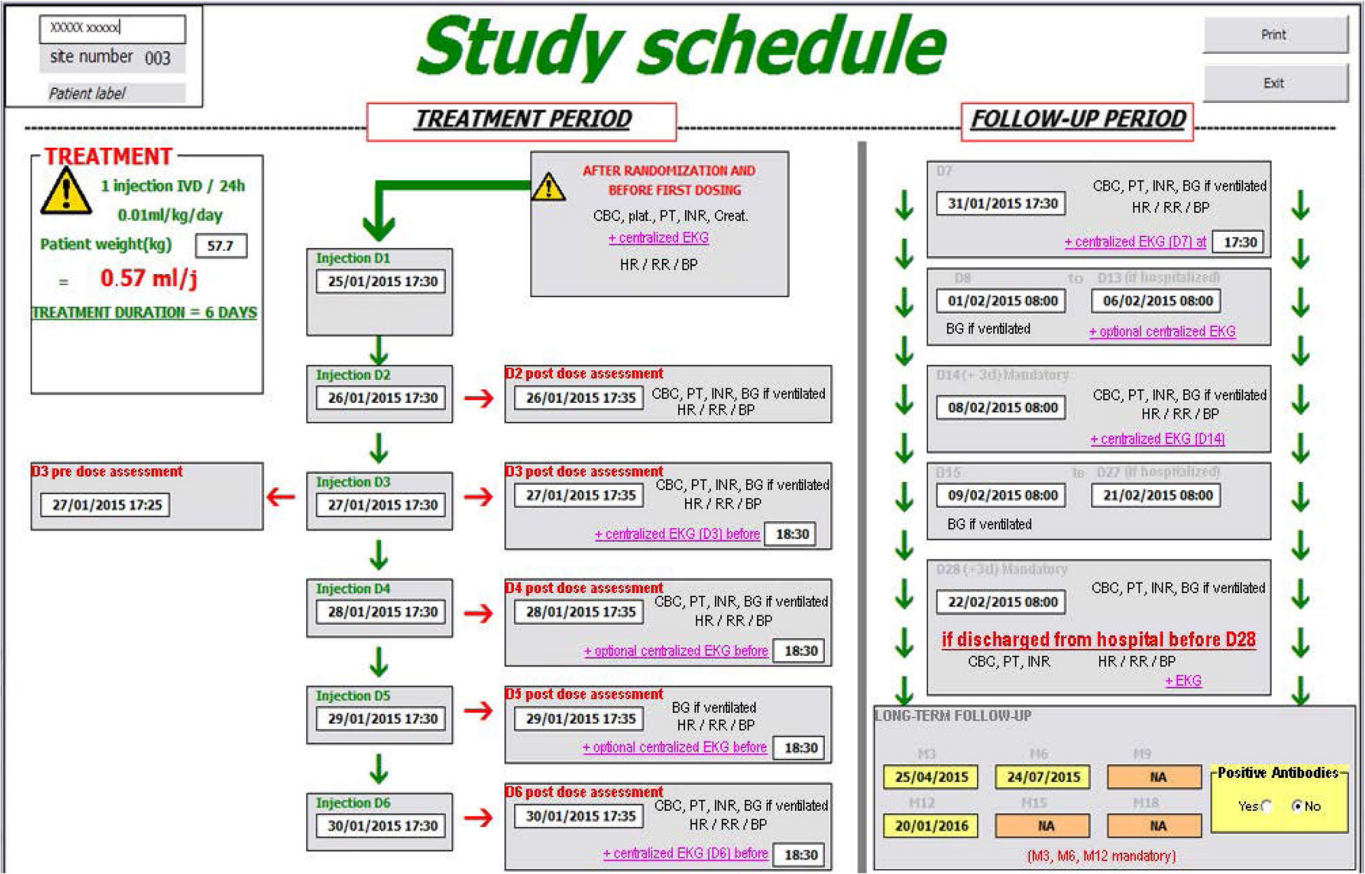
Example of a “schedule of event” for bedside nurses in a sepsis trial. It includes both treatment and follow-up period until D28 or hospital discharge as well as long-term follow-up. Treatment dose is automatically calculated based on patient’s weight and date and time of treatment and/or assessment are automatically displayed based on the time of inclusion. Each assessment box provides information on data to record and examination to perform. *CBC* complete blood count, *plat.* platelet count, *PT* prothrombin time, *INR* international normalized ratio, *Creat* creatinine, *EKG* electrocardiogramme, *HR* heart rate, *RR* respiratory rate, *BP* blood pressure, *BG* blood gas, *NA* not applicable

Over the last 20 years, ICU took benefit of the technological revolution with a very large use of computerized and monitoring systems. Clinical research has incorporated these changes from generalization of eCRF to trial management system through centralized and computerized randomization systems. Nevertheless, very little has been made to “computerize” investigation and to develop tools facilitating investigators tasks. Optimization of RCT can start with the integration of all study specificities (e.g., drug traceability or specific biological sampling) in the computerized medical record and monitoring system. Nevertheless, some more practical instruments can be developed especially for patient screening, such as lab alerts plugged on the hospital biology results, automated notification systems for potential patients [47] or even screening tools (Fig. 2) that help the investigator at bedside but also confirm patient eligibility. After patient enrollment, schedule of event of ICU trials is usually cumbersome with very precise timing and follow-up 24/7. Accordingly, development of scheduling tools for bedside nurses can make acceptance of research easier but also avoid any omission or delay that could be harmful to both the patient and the trial (Fig. 3). For the long-term follow-up (sometimes 1 year) which is currently commonly required in trials, helping tracking tools with automatic reminder can also be developed in order not to miss any visit and avoid as much as possible loss of follow-up. This computerized approach will undoubtedly blow up in the coming years with pocket system or even with applications directly developed for smartphones [48].

#### Big data and bioinformatics

As stated previously, ICUs have now implemented many computerized and monitoring systems producing a huge quantity of data. To date, the data are mainly collected for patient routine care or for a specific purpose such as a trial and are not used “outside.” This is progressively changing and big data is currently being implemented at patients’ bedside and in clinical research. For example, “fused parametric measures” are routinely used to determine the level of severity [49]. In clinical trials, “pooled trial data” seems to be an inevitable evolution that might be very useful in the future. Datasets from existing clinical trials would be pooled to perform secondary analysis in order to acquire new knowledge or reveal trends that would have been missed in smaller datasets [50]. Pooled data could also be used to design the studies, for example to calculate more accurately the sample size or to identify the current problems that cause failure. Finally, big data could also be implemented directly in the process of drug discovery. In oncology, this aspect is currently being investigated as physiologic information from national databases are compared to treatment information in order to connect specific phenotypes to molecular activity [51]. To date, the major limitation to big data implementation is the absence of ethical and legal context but some guidelines are already being published [50].

#### Training

In most of countries, physicians do not receive specific clinical research training during their medical training. Usually, physicians start with basic science and sometimes move to clinical investigation mainly when they join a department participating in RCTs [52]. Most of the training is usually acquired through e-learning provided by pharmaceutical sponsors or CROs and mainly focused on GCP. Although GCPs are widely considered the standard background for participating in RCTs, it only covers a few aspects of clinical research. Thus, most of the investigators, especially in pharmaceutical-sponsored RCTs, are only involved in recruitment with little participation in the rest of the trial. In order to optimize the conduction and completion of clinical trials, physicians have to become more than “simple investigators” through specific training course, visiting all aspects of clinical research including methodology, trial management, pharmacovigilance, monitoring, audit, drug supplying, CRF… A perfect knowledge and understanding of clinical research will take part in the necessary professionalization of this activity. This training should become part of the medical studies but in the meantime, training of all the human resources of investigation sites is key, involving the physicians but also the nurses and other staff members that have usually less medical expertise.

#### Defining new collaborations with pharmaceutical companies

Since a long time, clinical investigation with pharmaceutical companies has been developed through site sub-contracting processes. Investigators are usually not involved in the trial design or management; most of these responsibilities being held by the steering committee and CROs are usually responsible for the overall trial management on behalf of the sponsor. This model should probably be revised, especially in the ICU setting because of its huge specificity with a deeper participation of academic investigators in trial design, set-up of different committees, and trial management [53]. This would create a more collaborative research environment and increase site commitment. This paradigm shift is necessary in a period when lots of ICU professionals complain about trial realization. It could participate undoubtedly in a general improvement of RCT delivery. Some innovative experiences are currently developed in different countries and especially in Europe with the IMI collaboration [54] but also in the USA.

#### Comprehensive approach

From now on, clinical research in the ICU should not be any more considered as a stand-alone activity on top of routine patient care when people are available. The true change to perform to improve clinical research is to have a comprehensive approach, as it becomes part of patient care like in Oncology, and is not only an optional activity. While for years, treatment approaches have been very heterogeneous from site to site, standardization has started few years ago and nowadays, critical care medicine benefits from several international treatment recommendations, the most popular being the Surviving Sepsis Campaign [55]. In the meantime, a lot of universal definitions for the ICU-specific diseases have been validated, for care and research purposes. As an example, in 1992, the American College of Chest Physicians/Society of Critical Care Medicine Consensus Conference issued definitions regarding sepsis and the use of innovative therapies [3]; the Food and Drug Administration (FDA) has published recommendations and definition for VAP trials [56]. Accordingly, for sepsis and VAP, every patient wherever treated in the world should receive standard treatment and systematically be considered for eligibility in RCTs to potentially improve outcome and fill national and international database like for cancer [57] or cardiology patients. Continuous results from RCTs for these ICU topics with epidemiological prospective information will not only help understanding the diseases but also improve their management and thus outcome. Nevertheless, this requires a complete revolution at the site level in order to make clinical research part of routine care involving all staff members from the secretary to the physicians and the nurses. This is not an easy milestone in the ICU setting, its intrinsic specificities (emergency, life-threatening condition, ethical issues, number of players…) pushing back against it.

The problems identified and the potential solutions are summed up in Table 1.

**Table 1:** Summary of issues identified and proposed solutions

Issues identified	Potential consequences for the study	Proposed solutions
Weakness of definitions of ICU diseases	Difficult to demonstrate trial and/or drug efficacy	Increase basic research, for example, on biomarkers
Current trial designs unadapted to ICU specificities	Lower impact of results and efficacy of trial	Adaptive design Personalized medicine
80 % of patients recruited by 20 % of sites	Possible bias, center effect	Improved site selection
Overestimation of recruitment capacity	Recruitment objectives not met, study closed	More precise calculation of recruitment capacity Evaluation of prior performance
Competing trials not considered during feasibility	Patients eligible for several studies	Tracking tables of all studies even potential
Caregivers not involved in research	Possible bias due to lack of information	Involvement of every member of the unit
Numerous players	Confusion, delay or lack of information	Standard operating procedures with different units Regular meetings
Under recruitment	Study closed for lack of patients	Research team available 24/7 Systematic screening procedures Clinical coordinating center
Complex and tight schedule of events	Missing data, delay	Computerized medical records Automatic alerts for results or treatment
Long-term follow-up of patients	Many patients lost to follow-up	Tracking tables with reminders
Lack of training for physicians	Physicians do not feel involved	Training included as soon as medical studies Specific training courses
Lack of involvement of investigators in pharmaceutical-sponsored trials	Design unadapted to ICU research Investigators do no feel involved	Involvement of physicians since the beginning of the process
Clinical research still considered as a stand-alone activity	Cares and treatments competing with routine	Clinical research implemented as part of routine care

## Conclusions

Clinical research has become a true activity that not only provides new medical knowledge or brings validation of new drugs but also improves patient care through very high standard of quality and rigorous delivery. Because of its increasing requirements, it needs a global approach made of various competencies and professionalization. In this environment, ICU remains a very unique medical field with numerous specificities related to the type of admitted patients and to the 24/7 medical activity. This requires developing specific and optimized approach for clinical research in critical care medicine to create its own identity. The investigators have to make themselves the leaders of this transformation. Time for paradigm shift in clinical research in the ICU has come.

## Abbreviations

ARDS: Acute respiratory distress syndrome
CCC: Clinical coordinating center
CRF: Case report form
CRO: Clinical research organization
FDA: Food and Drug Administration
GCP: Good clinical practice
ICH: International Harmonization Conference
ICU: Intensive care unit
RCT: Randomized clinical trial
VAP: Ventilator-associated pneumonia
